# Differential expression of protein disulfide-isomerase A3 isoforms, PDIA3 and PDIA3N, in human prostate cancer cell lines representing different stages of prostate cancer

**DOI:** 10.1007/s11033-021-06277-1

**Published:** 2021-03-24

**Authors:** Maria Araceli Diaz Cruz, Sandra Karlsson, Ferenc Szekeres, Maria Faresjö, Dan Lund, Dennis Larsson

**Affiliations:** 1 Research School of Health and Welfare, School of Health and Welfare, University of Jönköping, Jönköping, Sweden; 2 Department of Natural Science and Biomedicine, School of Health and Welfare, University of Jönköping, Jönköping, Sweden; 3 Department of Biomedicine, School of Health Sciences, University of Skövde, Skövde, Sweden; 4 Sahlgrenska University Hospital, Gothia Forum - for Clinical Research, Gothenburg, Sweden

**Keywords:** Prostate cancer, Vitamin D, PDIA3, PDIA3N, VDR, Androgen dependency

## Abstract

Prostate cancer (PCa) is a highly heterogeneous and unpredictable progressive disease. Sensitivity of PCa cells to androgens play a central role in tumor aggressiveness but biomarkers with high sensitivity and specificity that follow the progression of the disease has not yet been verified. The vitamin D endocrine system and its receptors, the Vitamin D Receptor (VDR) and the Protein Disulfide-Isomerase A3 (PDIA3), are related to anti-tumoral effects as well as carcinogenesis and have therefore been suggested as potential candidates for the prevention and therapy of several cancer forms, including PCa. In this study, we evaluated the mRNA expression of VDR and PDIA3 involved in vitamin D signaling in cell lines representing different stages of PCa (PNT2, P4E6, LNCaP, DU145 and PC3). This study further aimed to evaluate vitamin D receptors and their isoforms as potential markers for clinical diagnosis of PCa. A novel transcript isoform of PDIA3 (PDIA3N) was identified and found to be expressed in all PCa cell lines analyzed. Androgen-independent cell lines showed a higher mRNA expression ratio between PDIA3N/PDIA3 contrary to androgen-dependent cell lines that showed a lower mRNA expression ratio between PDIA3N/PDIA3. The structure of PDIA3N differed from PDIA3. PDIA3N was found to be a N-truncated isoform of PDIA3 and differences in protein structure suggests an altered protein function i.e. cell location, thioredoxin activity and affinity for 1,25(OH)_2_D_3_. Collectively, PDIA3 transcript isoforms, the ratio between PDIA3N/PDIA3 and especially PDIA3N, are proposed as candidate markers for future studies with different stages of PCa progression.

**Supplementary Information:**

The online version contains supplementary material available at 10.1007/s11033-021-06277-1.

## Introduction

Prostate cancer (PCa) is one of the most common cancer types worldwide and the second leading cause of death among men in the United States and Europe [1, 2]. The disease is heterogeneous and unpredictable since prostate cancer cells easily can pass from indolent to rapidly progressive and may lead to fatality [3].

A major clinical challenge in PCa screening is the lack of diagnostic tests that distinguish between benign and aggressive tumors [1, 4]. Standard approaches used are tissue pathology and Gleason score, imaging and prostate specific antigen (PSA) serum levels [1–3, 5]. Analyses of tissue pathology and Gleason score classifications are more reliable but require invasive techniques to extract biopsies from the tumor. Imaging is not used if tumor growth is not expected and usually miss small tumors. PSA screening is the most effective non-invasive method used for more than 20 years. However, PSA screening has low sensitivity and marginal specificity resulting in high number of false positives associated with other uropathies [4]. Thus, there is a need of finding candidate biomarkers with both high sensitivity and specificity to follow PCa progression.

New gene transcript isoforms are emerging as suitable candidates to represent disease progression since high concentrations of specific protein isoforms may be associated with specific tumor stages or level of disease progression [6, 7]. An example is the discovery of an alternative splicing isoform signature associated with overall survival for hepatocellular carcinoma [8]. Also, the isoform switches are highly predictive for cancer survival and aggressiveness, such as the DNA excision repair 1 (ERCC1) gene resulting in a protein lacking the HHH domain associated with lower cancer survival rates [9].

Vitamin D and its metabolites have been suggested as potential candidates for the prevention and therapy of several cancer forms, including PCa [10, 11]. Numerous reports demonstrate that vitamin D, through 1,25-dihydroxyvitamin D_3_ (1,25(OH)_2_D_3_), exerts antitumor effects. 1,25(OH)_2_D_3_ has been demonstrated to stimulate differentiation, increase apoptosis and inhibit proliferation, invasiveness and metastasis of cancer cells [11, 12]. Two receptors are known to be involved in the vitamin D signaling pathway and its mediated effects. The nuclear vitamin D receptor (VDR) and the protein disulfide isomerase family A, member 3 (PDIA3) [13–15]. VDR is localized in the cytosol and becomes activated upon binding with 1,25(OH)_2_D_3_, leading to a heterodimerization with the RXR receptor. This complex migrates to the nucleus where it modulates gene transcription after binding with the vitamin D response element (VDRE) [16, 17]. PDIA3 is a chaperone protein localized mainly in the endoplasmic reticulum, suggested to be responsible for “rapid actions” of 1,25(OH)_2_D_3_ initiated at the plasma membrane [13, 14]. These actions include regulation of intracellular, extranuclear pathways and signaling cascades, such as the activation of the protein kinase C (PKC) pathway and the calcium transport [13]. The role of PDIA3 in cancer regulation remains controversial since some studies suggest that it is responsible for the activation of proapoptotic pathways [18, 19] whereas others suggest association with cancer proliferation, inhibition of apoptosis and poor prognosis [20, 21]. In both normal and cancer prostate tissues, VDR is found to be lower both at level of transcription and translation compared to PDIA3 [22].

We evaluated mRNA expression of VDR and PDIA3 involved in vitamin D signaling in cell lines representing different stages of PCa (PNT2, P4E6, LNCaP, DU145 and PC3). Further, we aimed to evaluate vitamin D receptors and their isoforms as potential markers for clinical diagnosis of PCa.

## Materials and methods

### In vitro cell culture

Following commercial prostate cells, representing different stages of prostate cancer, were used in the present study (Supplementary File 1); PNT2, P4E6 and LNCaP (Sigma Aldrich, St. Louis, MO, USA), DU145 and PC3 (ATCC, Manassas, VA, USA). PNT2 and LNCaP cells were cultured in RPMI-1640 supplemented with 10% FBS and 1% PEST (Sigma Aldrich), whereas P4E6 cells were maintained in Stemline Keratinocyte Medium II with Stemline Keratinocyte Growth Supplement, 2 mM Glutamine and 2% of FBS (Sigma Aldrich), DU145 cells were cultured in EMEM (Sigma Aldrich) supplemented with 10% FBS and 1% PEST and PC3 cells were cultured in DMEM (Sigma Aldrich) containing 10% FBS, 5% of pyruvate sodium (Sigma Aldrich) and 1% PEST.

### RNA extraction and reverse transcription (RT-PCR)

Total RNA was extracted from approximately 1 × 10^6^ cells, using RNeasy Mini Kit including genomic DNA digestion with DNase (Qiagen, Hilden, DE). High quality RNA (A260/280 = 1.9–2.0, A260/230 = 1.5–1.8, RNA integrity number ≥ 8) from the respective cell line was sent for NGS analysis to The National Genomics Infrastructure in The Science Life Lab in Stockholm, Sweden (NGI, SciLifeLab).

For digital droplet PCR preparation, RNA (up to 2 µg) extracted from PNT2, DU145, PC3 and LNCaP cells was reversed transcribed to cDNA according to the manufacturer´s protocol for the High Capacity cDNA Reverse Transcription Kit (Applied Biosystems, Foster City, CA, USA).

### New generation sequencing (NGS)

A total of 20 ug of RNA from PNT2, P4E6, DU145, PC3 and LNCaP cells were sequenced with Illumina RNAseq HiSeq 2500 High Output v4, 2 × 125 bp in one lane giving > 37.6 M read pairs/sample.

Software TopHat/2.0.4 was used for mapping reads to the Human genome assembly, build GRCh37 (hg19). Quantification of normalized expression genes and their different transcripts isoforms were obtained as FPKM (Fragments per Kilobase of Exon per Million Fragments Mapped) generated by cufflinks/2.1.1.

### Droplet digital ™ PCR (ddPCR)

Droplet digital polymerase chain reaction was performed according to the manufacturer´s protocol (ddPCR; QX200, Bio-Rad, Hercules, CA, USA). The ddPCR assays, consisting of two primers and TaqMan hydrolysis probe for each target, were designed using Primer3 Plus web interface [23] (Supplementary File 2). A total of 225 cDNA samples (5 cell lines, 3 biological samples × 3 different passage numbers and 5 technical replicates for each sample) were analyzed to quantify the amount of PDIA3-Normal and PDIA3-Novel target. The amount of cDNA for each reaction was 10,000 copies corresponding to 33 ng of DNA. Each sample was partitioned into 10,000–18,000 droplets and transferred to a 96-well-plate for subsequent PCR amplification.

After amplification, droplets were read on a QX200 droplet reader (Bio-Rad) and analyzed with QuantaSoft software V1.7.4 (Bio-Rad). Amplitude limit was set manually to 2000 for PDIA3 and 4000 for PDIA3N. The Poisson-corrected determination of mRNA expression or template concentration (copies/ µl) and the ratio of mRNA expression between the isoforms (PDIA3-Novel/PDIA3-Normal) were calculated using QuantaSoft™ Analysis Pro Software (v1.0.596, Bio-Rad). As validation, the same experiment was repeated using 45 randomly selected samples.

### Statistical analysis

Nonparametric tests were used to determine differences in mRNA expression between PCa cell lines assuming that the data did not follow a normal (Gaussian) distribution (Kolmogorov–Smirnov test and D’Agostino & Pearson test, P value < 0.0001). Differences between ratios of mRNA expression among PCa cell lines were assessed by non-parametric Kruskal–Wallis multiple comparison test. Pairwise comparison between PCa cell lines were assessed by Mann–Whitney *U*-test. Pairwise comparisons in the same PCa cell line were assessed with Wilcoxon signed rank test. All statistical tests were performed with GraphPad Prism version 8.3 for Windows (GraphPad Software, La Jolla, CA, USA). Levels of significance were set to *: P < 0.05, **: P < 0.01, ***: P < 0.001 and ****: P < 0.0001.

### Functional analysis

Protein sequences were retrieved from UniprotKB (P30101) for PDIA3 and from UniParc (UPI000066D935) for PDIA3N [24]. Both protein sequences were analyzed by I-TASSER (Iterative Threading ASSEmbly Refinement server) to generating the protein structure model [25]. Differences in protein sequences were highlighted with UCSF Chimera 1.0 [26]. A prediction of damage or pathogenicity of the 56 variations (amino acid substitutions and deletions) that PDIA3N present in comparison with PDIA3 was carried out with PolyPhen-2 [27] and PROVEAN (Protein Variation Effect Analyzer) v1.1 [28]. A prediction of subcellular localization of PDIA3 and PDIA3N was performed by DeepLoc-1.0 [29].

To address differences in 1,25(OH)_2_D_3_ binding with PDIA3 and PDIA3N, an automated docking of ligand to macromolecular receptor, without allowing flexible conformation, was performed with Autodock 4.0 software and analyzed with Autodock tools (ADT) [30]. Input coordinates of 1,25(OH)_2_D_3_ were extracted from the crystal structure of VDR complexed bound to 1,25(OH)_2_D_3_ [31]. Grid spacing was set to 0.375 Å, number of energy evaluations was set to 2.5 × 10^6^ and 150 as population size for PDIA3 and VDR and 5 × 10^6^ and 500 for PDIA3N, respectively. Putative binding sites in PDIA3 and PDIA3N were selected by searching for free binding energies similar to the obtained in previous experiments with VDR and 1,25(OH)_2_D_3_ [15, 32].

## Results

**Fig. 1:**
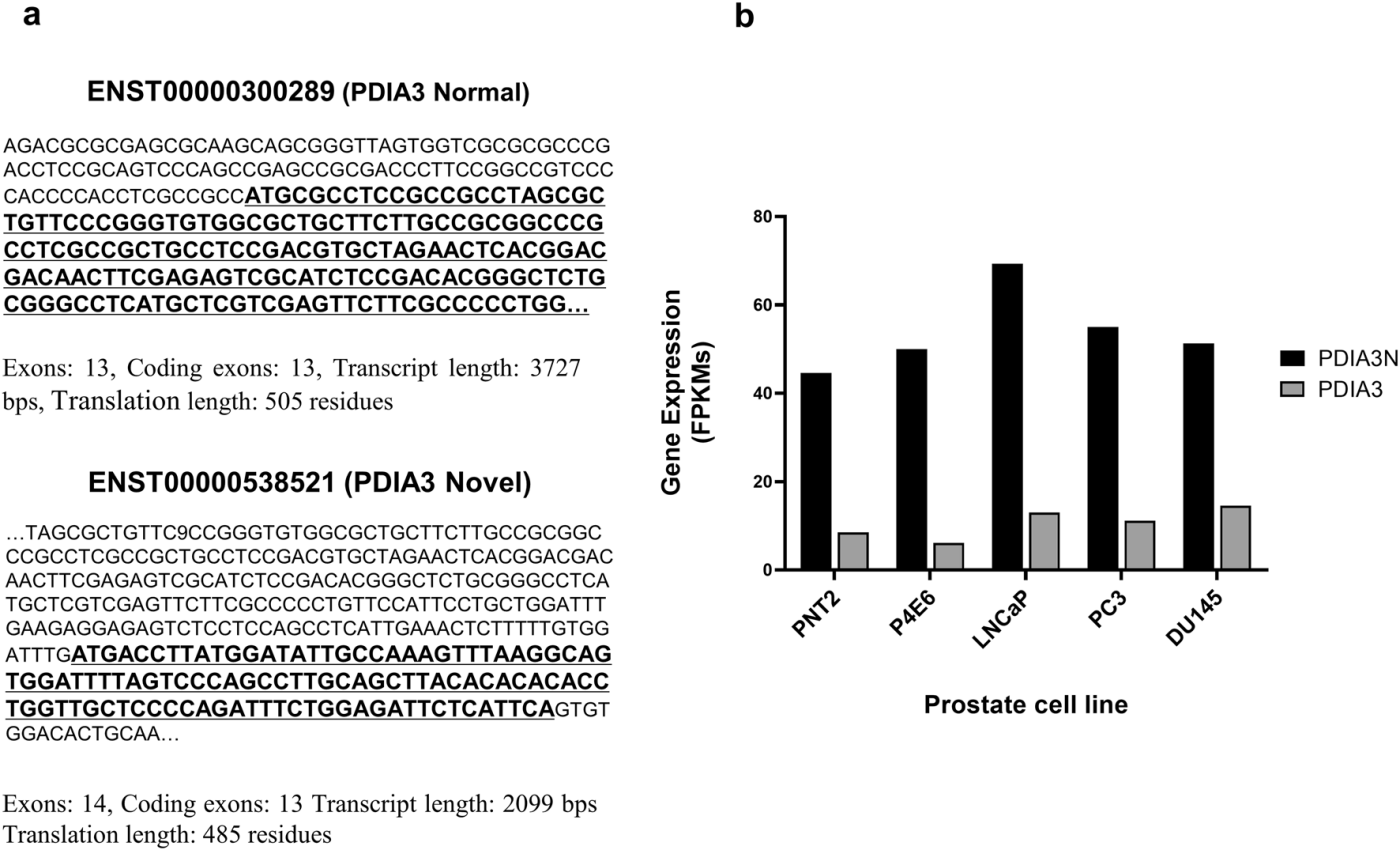
NGS results from prostate cell line RNA samples. **a** Differences in DNA sequence for the PDIA3 normal transcript isoform and PDIA3 Novel transcript isoform. **b** PDIA3 and PDIA3N expression values (FPKM) obtained after cufflinks/2.1.1 software analysis of NGS results for prostate cell lines PNT2, P4E6, DU145, PC3 and LNCaP

Five prostate cell lines, PNT2, P4E6, DU145, PC3 and LNCaP, were analyzed by NGS and all these cells lines showed low mRNA expression of VDR and RXRA (0–7.4 FPKMs). A novel transcript isoform of PDIA3 (PDIA3N, ENST00000538521.1, GRCh37.p13 Ensembl 2018) [33] was also detected in all five prostate cell lines (Fig. 1A). PDIA3N was found to be 1628 bp shorter than PDIA3 (ENST00000300289, GRCh38.p12 Ensembl 2018 [33] and contained a different nucleotide sequence fragment of 178 bp, which included an alternative translation initiation site (TIS; secondary starting codon “ATG”, Fig. 1a). PDIA3N had higher mRNA expression compared to PDIA3 in prostate cells with normalized expression values of 8.5–14.5 and 44.6–69.3 FPKM, respectively (Fig. 1b). Expression of PDIA3N was correlated to tumor stage, being more expressed in metastatic cell lines (DU145, PC3 and LNCaP) compared to cell lines derived from normal prostate tissue (PNT2) and the cell line derived from an early stage of androgen negative prostate cancer (P4E6). The highest expression of PDIA3N (69.3 FPKM) was obtained from the metastatic androgen dependent cell line LNCaP (Fig. 1b).

**Fig. 2:**
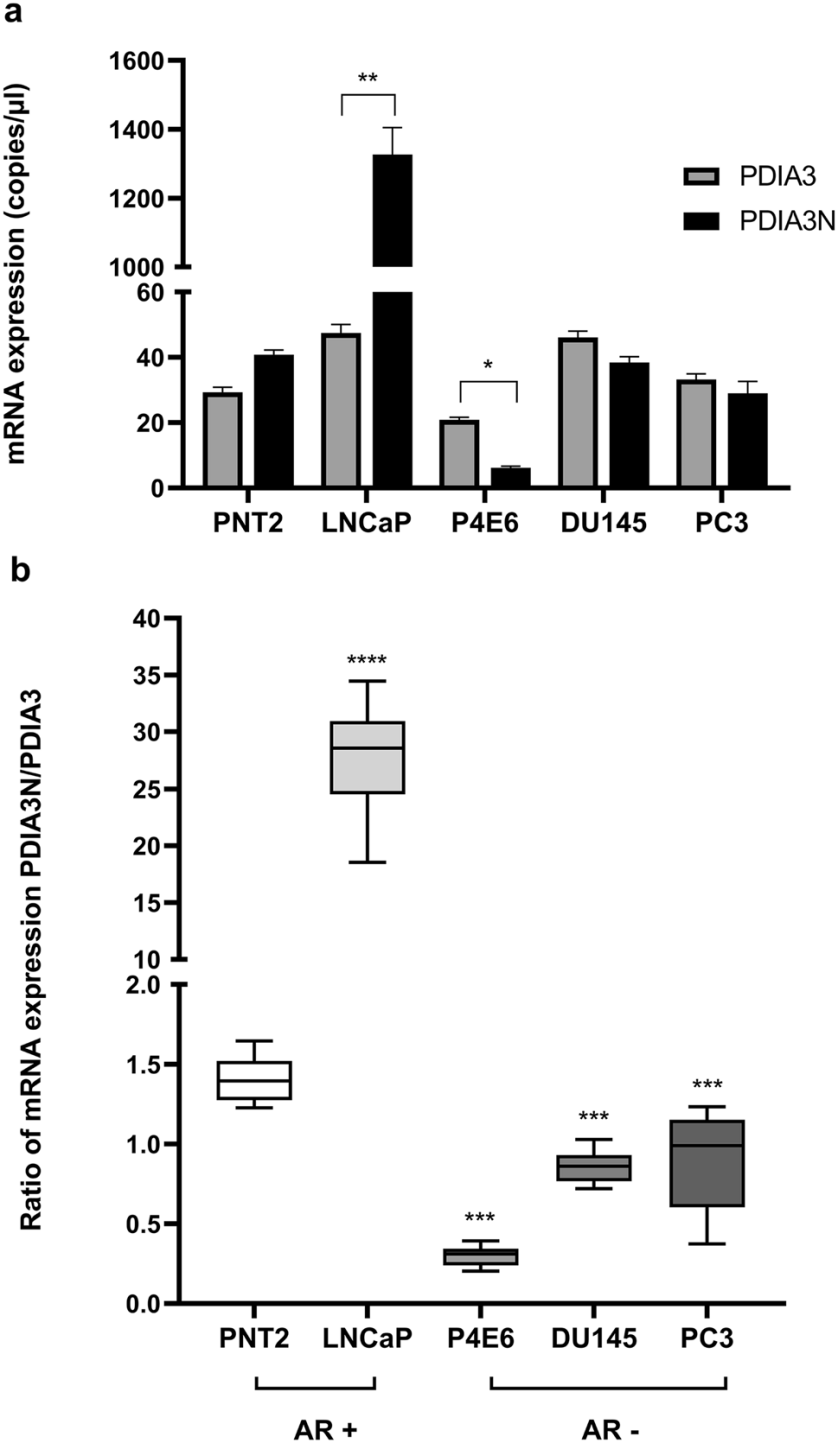
Results from ddPCR absolute quantification experiments of cDNA samples from androgen receptor positive (AR +) cells, PNT2 and LNCaP; and androgen receptor negative (AR-) cells, P4E6, DU145 and PC3. **a** The mRNA expression (copies/ul) of PDIA3 and PDIA3N isoforms, and **b** the ratio of PDIA3N/PDIA3 mRNA expression

**Fig. 3:**
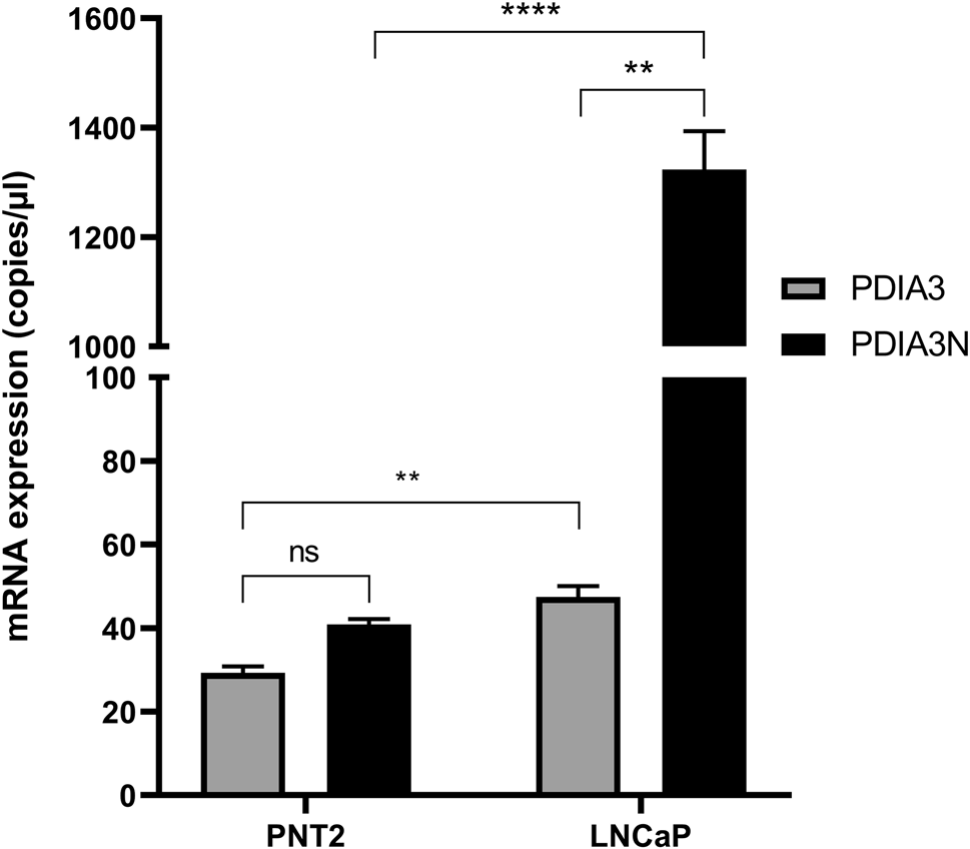
Amplification results from ddPCR experiment showing PDIA3 and PDIA3N mRNA expression (copies/ µl) between the normal epithelial cell line PNT2 and the androgen-dependent metastatic cell line LNCaP

Dependent on the findings in the NGS analyses of a novel isoform of PDIA3, mRNA expression of PDIA3 and PDIA3N were further evaluated by ddPCR in PNT2, P4E6, DU145, PC3 and LNCaP cells. Among AR positive cell lines, LNCaP showed a higher mRNA expression of PDIA3N compared to PDIA3 (p < 0.01), whereas PNT2 showed a similar trend (p = 0.063). Contrary to AR positive cell lines, AR negative cell lines P4E6 (p < 0.05), DU145 (0.063) and PC3 (0.69) showed a lower or an equally high mRNA expression of PDIA3N compared to PDIA3 (Fig. 2a). AR negative cell lines (P4E6, DU145 and PC3) showed lower PDIA3N/PDIA3 ratio compared to AR positive cell lines (PNT2 and LNCaP) (p < 0.0001). PDIA3N/PDIA3 was higher in the metastatic cell line LNCaP compared to the normal epithelial cell line PNT2 (p < 0.0001, Fig. 2b). PDIA3N/PDIA3 was lower in P4E6, DU145 and PC3 compared to the normal epithelial cell line PNT2 (p < 0.001, Fig. 2b). Expression of PDIA3 and PDIA3N was higher in LNCaP cells compared to PNT2 cells (p < 0.001 and p < 0.0001 respectively; Fig. 3). Expression of PDIA3N in LNCaP cells was higher compared to PDIA3 (p < 0.0001; Fig. 3).

**Fig. 4:**
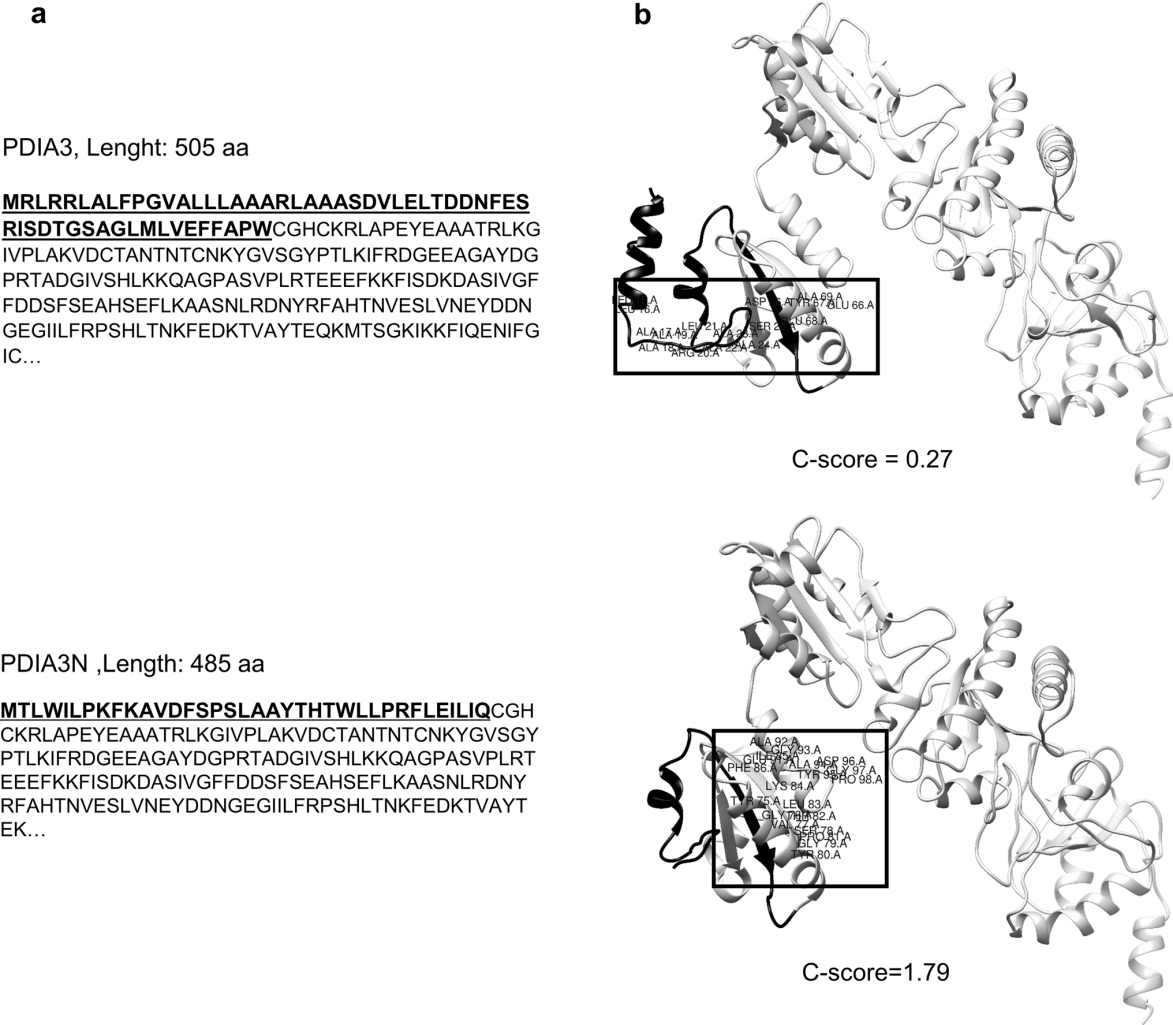
Predicted protein structure of PDIA3 and PDIA3 novel isoform analyzed with I-TASSER. **a** Protein sequence for PDIA3 (Top) and PDIA3N (Bottom). **b** Structure prediction pdb model of PDIA3 (Top) and PDIA3N (Bottom) visualized in UCSF Chimera. The protein sequence that differs between both isoforms is highlighted in black and the amino acids that conform the 1,25(OH)_2_D_3_ binding pocket are labelled and marked with a rectangle in (**b**)

A generated protein structure model for PDIA3 and PDIA3N are shown in Fig. 4. The protein sequence for PDIA3N contains 485 amino acids. The first fragment of the N-terminus differs in 56 amino acids (36 amino acid substitutions and 20 amino acid deletions) from PDIA3 (Fig. 4a). PDIA3N had a higher confidence (C-Score) than PDIA3 (Fig. 4b, I-TASSER). At the secondary structure level, PDIA3N lacks an α-helix. Furthermore, PDIA3N contains a truncated thioredoxin site in the N-terminus (CGH from WCGH, Fig. 4a, b). Twenty-two out of the 56 altered amino acids in the N-terminus of PDIA3N were confirmed to affect the structure and function of the protein (PolyPhen-2 and PROVEAN, Supplementary File 3). Analysis of subcellular localization for PDIA3N and PDIA3 showed PDIA3N to be present in the cytosol while PDIA3 is present in the endoplasmatic reticulum (ER) (Supplementary File 4). Binding capacity for PDIA3 and PDIA3N to 1,25(OH)_2_D_3_ revealed a putative binding site in the N-terminus with an estimated free energy of binding of − 9.29 kcal/mol (estimated inhibition constant, Ki = 155 nM) and -8.21 kcal/mol (Ki = 958 nM, respectively) (Supplementary File 5).

## Discussion

Isoforms of gene transcripts are suggested as plausible biomarkers for different diseases, such as cancer. The expression pattern of different isoforms may change depending on the stage or severity of disease and relation between expression of two gene isoforms can be correlated with a specific disease pattern [9, 34, 35]. Previous studies pinpoint PDIA3 as a plausible candidate for studying cancer prognosis as a target for cancer treatment [18–21, 36]. The role of PDIA3 in cancer treatment remains controversial and implication of different PDIA3 transcript isoforms has not yet been studied in connection with progression of PCa.

In the present study, a novel PDIA3 transcript isoform was detected in prostate cell lines, in line with previous studies on kidney and colon cells where PDIA3N is associated with cancer progression [24, 33]. Furthermore, PDIA3N has a higher mRNA expression compared to PDIA3 in prostate cells with especially high mRNA expression in LNCaP cells. Interestingly, this study indicates for a correlation between the ratio of PDIA3N/PDIA3 and cancer stage. The structure of PDIA3N differed from PDIA3 suggesting an altered function of the protein concerning cell location, thioredoxin activity and affinity for 1,25(OH)_2_D_3_. Thus, a shift in ratio between PDIA3N and PDIA3 may contribute to cancer progression by decreased redox activity and altered hormone function. Furthermore, the ratio between PDIA3N and PDIA3 might be a relevant indicator to follow in future studies with focus on progression of PCa.

Several studies associate changed expression of PDIA3 with multiple pathologies including cancer and neurodegenerative disease [36–38]. Aberrant expression of PDIA3 is shown to be correlated with poor prognosis in several cancer types and increased cell proliferation mediated by 1,25(OH)_2_D_3_ and subsequent activation of the epidermal growth factor receptor (EGFR) [36]. So far only one study has evaluated expression of PDIA3 in PCa tissue in which tumor samples with a higher Gleason score (GS 8–10) had higher expression of PDIA3 compared to GS 6 tumors [18]. However, a difference in PDIA3 expression between GS 8–10 and benign tumor tissue was not observed [18]. Our result is in line with previous observations [23] suggesting PDIA3 as a key protein that might contribute to cancer development.

An imbalance in ratio between VDR and PDIA3 (including PDIA3 isoforms) might contribute to PCa progression, as suggested in this study. An increased pool of PDIA3N would bind a large part of available 1,25(OH)_2_D_3_ and prevents, in part, the hormone to interact with VDR. However, VDR has a higher affinity to 1,25(OH)_2_D_3_ (Ki of 1–10 nM) [15] compared to PDIA3 (Ki of 155 nM) and PDIA3N (Ki of 958 nM). This indicate that 1,25(OH)_2_D_3_ may preferentially bind to VDR but the larger pool of PDIA3 and PDIA3N will bind a considerable part of available 1,25(OH)_2_D_3_. Taken together, we suggest that the decreased availability of 1,25(OH)_2_D_3_ to VDR and lower levels of VDR have a negative impact on the antitumorigenic effects of 1,25(OH)_2_D_3_. The role of PDIA3N remains unclear but it may have a relevant role in pathogenesis due to its abundant expression in the metastatic androgen-dependent stage, compared to PDIA3, and due to its predicted aberrant structure and function. Thus, this study suggests that the different PDIA3 isoforms might play contrary roles in PCa progression. The switch in expression between these isoforms to a higher abundance of PDIA3N might indicate for a more severe stage of the disease.

The predicted protein sequence reveals that PDIA3N is shorter in length and contains a different N-terminus sequence than PDIA3 due to an alternative 5′-proximal translation initiation site (TIS). It has been shown that several TIS can be recognized by the ribosomes in the coding sequence (CDS) at the same time. This mechanism is called “leaky scanning” and is responsible for the translation of functional different isoforms [39, 40]. Most of these isoforms are N-truncated proteins that contain secretory signals in order to be delivered to different cell compartments [40]. The TIS discovered in PDIA3N leads to a different protein sequence in the N-terminus and indicates that this isoform is mainly present in the cytosol, a different subcellular cell compartment compared to PDIA3 which in a major part is found in the ER and suggests a different function.

Normally, the major part of PDIA3 is associated with the ER, forming a complex with calreticulin and calnexin, participating in correct folding and in quality control of neo-synthesized glycoproteins to be secreted or to be localized to the cell membrane [32, 41–43]. According to sequence analyses in the present study, a major part of the PDIA3 are present in the cytosolic compartment. Previous studies have demonstrated that PDIA3 forms complexes with the retinoic acid receptor [44], STAT3 [45–47], NF-κB [48] and Ref-1 [49] in the cytosol. Considering the thioredoxin sites and redox regulating capacity of PDIA3, it might impact the activity of complex bound proteins as well as regulating the redox potential within the cell. The predicted functional differences of PDIA3N compared to PDIA3, points out, according to our analyses, a cytosolic location. The structural change where one α-helix is missing in PDIA3Ns secondary structure and its truncation in one active thioredoxin site indicate for an impaired thioredoxin activity and might be the cause of a lower binding affinity to 1,25(OH)_2_D_3_. The functional differences of PDIA3N might in turn lead to impaired functions of the complexes formed with PDIA3N in the cytosol.

Altogether, these data suggest that the novel transcript isoform of PDIA3, PDIA3N, is a putative candidate for studying progression of PCa. Future expression analyses of PDIA3 and PDIA3N in clinical samples, correlated to the Gleason score and androgen dependency stage of PCa, are necessary to determine if PDIA3N is a suitable biomarker for advanced PCa as well as the role of PDIA3, PDIA3N and the vitamin D endocrine system in regulating prostate cell biology.

## Supplementary Information

Below is the link to the electronic supplementary material.

Supplementary file1 (DOCX 23 kb)

Supplementary file2 (DOCX 22 kb)

Supplementary file3 (DOCX 25 kb)

Supplementary file4 (TIF 816 kb)

Supplementary file5 (DOCX 19 kb)

Supplementary file6 (7Z 65039 kb)

Supplementary file7 (7Z 394 kb)

Supplementary file8 (7Z 48071 kb)
