# Supplementary material for: Differential expression of protein disulfide-isomerase A3 isoforms, PDIA3 and PDIA3N, in human prostate cancer cell lines representing different stages of prostate cancer
Source: Mol Biol Rep. 2021 Mar 24;48(3):2429–36. doi: 10.1007/s11033-021-06277-1 (PMC8060222; doi:10.1007/s11033-021-06277-1)
Supplement: Supplementary file 1 — Supplementary file1 (DOCX 23 kb) [file 11033_2021_6277_MOESM1_ESM.docx]

Table 1. Prostate cell lines cultivated for the different experiments in the study.

| **Name** | **Source** | **Stage** | **AR protein** | **Androgen dependency** |
| --- | --- | --- | --- | --- |
| PNT2 | Prostate epithelial | Normal | Positive-low | Yes |
| P4E6 | Prostate epithelial | Early cancer | Negative | Not reported |
| LNCaP | Adenocarcinoma-lymph node | Metastatic | Positive-high | Yes |
| DU145 | Primary carcinoma-Brain | Metastatic (II) | Negative | No |
| PC3 | Adenocarcinoma-Bone (vertebral) | Metastatic  (IV) | Negative | No |
