# Supplementary material for: Differential expression of protein disulfide-isomerase A3 isoforms, PDIA3 and PDIA3N, in human prostate cancer cell lines representing different stages of prostate cancer
Source: Mol Biol Rep. 2021 Mar 24;48(3):2429–36. doi: 10.1007/s11033-021-06277-1 (PMC8060222; doi:10.1007/s11033-021-06277-1)
Supplement: Supplementary file 2 — Supplementary file2 (DOCX 22 kb) [file 11033_2021_6277_MOESM2_ESM.docx]

Table 2: Primer and probe cDNA sequences for PDIA3 and PDIA3N isoforms in absolute quantification experiments with ddPCR.

| **Target** | **Primer sequence** | **Probe sequence** |
| --- | --- | --- |
| PDIA3 | 5´-GTGTGGCGCTGCTTCTTG- 3´ (forward) | 5´-[FAM] GCCTCGCCGCTGCCTCCGAC-3´ |
|  | 5´-AAGAACTCGACGAGCATGAG-3´ (reverse) |  |
| PDIA3N | 5´-GGCAGTGGATTTTAGTCCCA- 3´ (forward) | 5´-[HEX]ACACACACACCTGGTTGCTCCCCAGA-3´ |
|  | 5´-CAAGTCTCTTGCAGTGTCCA-3´ (reverse) |  |
