# Supplementary material for: Differential expression of protein disulfide-isomerase A3 isoforms, PDIA3 and PDIA3N, in human prostate cancer cell lines representing different stages of prostate cancer
Source: Mol Biol Rep. 2021 Mar 24;48(3):2429–36. doi: 10.1007/s11033-021-06277-1 (PMC8060222; doi:10.1007/s11033-021-06277-1)
Supplement: Supplementary file 3 — Supplementary file3 (DOCX 25 kb) [file 11033_2021_6277_MOESM3_ESM.docx]

Table 3. PDIA3 sequence variations in PDIA3N, that are predicted as harmful or damaging for the protein structure and function by Polyphen-2 and PROVEAN.

| **Mutation** | **Polyphen-2 HumDiv Prediction-Score** | **Polyphen-2 HumVar Prediction-Score** | **PROVEAN Prediction-Score** |
| --- | --- | --- | --- |
| L15S | Probably damaging-0.999 | Probably damaging-0.998 | Deleterious-  (-2.841) |
| L16P | Probably damaging-0.994 | Probably damaging-0.840 | Deleterious-  (-2.534) |
| S25W | Probably damaging-0.999 | Probably damaging-0.995 | Deleterious-  (-3.646) |
| D26L | Probably damaging-1.0 | Probably damaging-1.0 | Deleterious-  (-8.089) |
| V27L | Probably damaging-0.999 | Probably damaging-0.998 | Deleterious-  (-2.596) |
| L28P | Probably damaging-0.999 | Probably damaging-0.980 | Deleterious-  (-5.534) |
| E29R | Possibly damaging-0.660 | Possibly damaging-0.518 | Deleterious-  (-3.547) |
| T31L | Possibly damaging-0.660 | Possibly damaging-0.480 | Deleterious-  (-3.609) |
| D32E | Probably damaging-0.975 | Probably damaging-0.927 | Deleterious-  (-3.203) |
| N34L | Possibly damaging-0.956 | Possibly damaging-0.882 | Deleterious-  (-4.518) |
| F35I | Probably damaging-1.0 | Probably damaging-1.0 | Deleterious-  (-5.266) |

| **Mutation** | **Polyphen-2 HumDiv Prediction-Score** | **Polyphen-2 HumVar Prediction-Score** | **PROVEAN Prediction-Score** |
| --- | --- | --- | --- |
| S37del | - | - | Deleterious-  (-4.275) |
| R38del | - | - | Deleterious-  (-4.842) |
| I39del | - | - | Deleterious-  (-3.643) |
| L49del | - | - | Deleterious-  (-3.824) |
| V50del | - | - | Deleterious-  (-6.236) |
| E51del | - | - | Deleterious-  (-8.939) |
| F52del | - | - | Deleterious-  (-12.045) |
| F53del | - | - | Deleterious-  (-12.045) |
| A54del | - | - | Deleterious-  (-12.826) |
| P55del | - | - | Deleterious-  (-15.312) |
| W56del | - | - | Deleterious-  (-18.703) |
