# Supplementary figures and images for: Differential expression of protein disulfide-isomerase A3 isoforms, PDIA3 and PDIA3N, in human prostate cancer cell lines representing different stages of prostate cancer
Source: Mol Biol Rep. 2021 Mar 24;48(3):2429–36. doi: 10.1007/s11033-021-06277-1 (PMC8060222; doi:10.1007/s11033-021-06277-1)

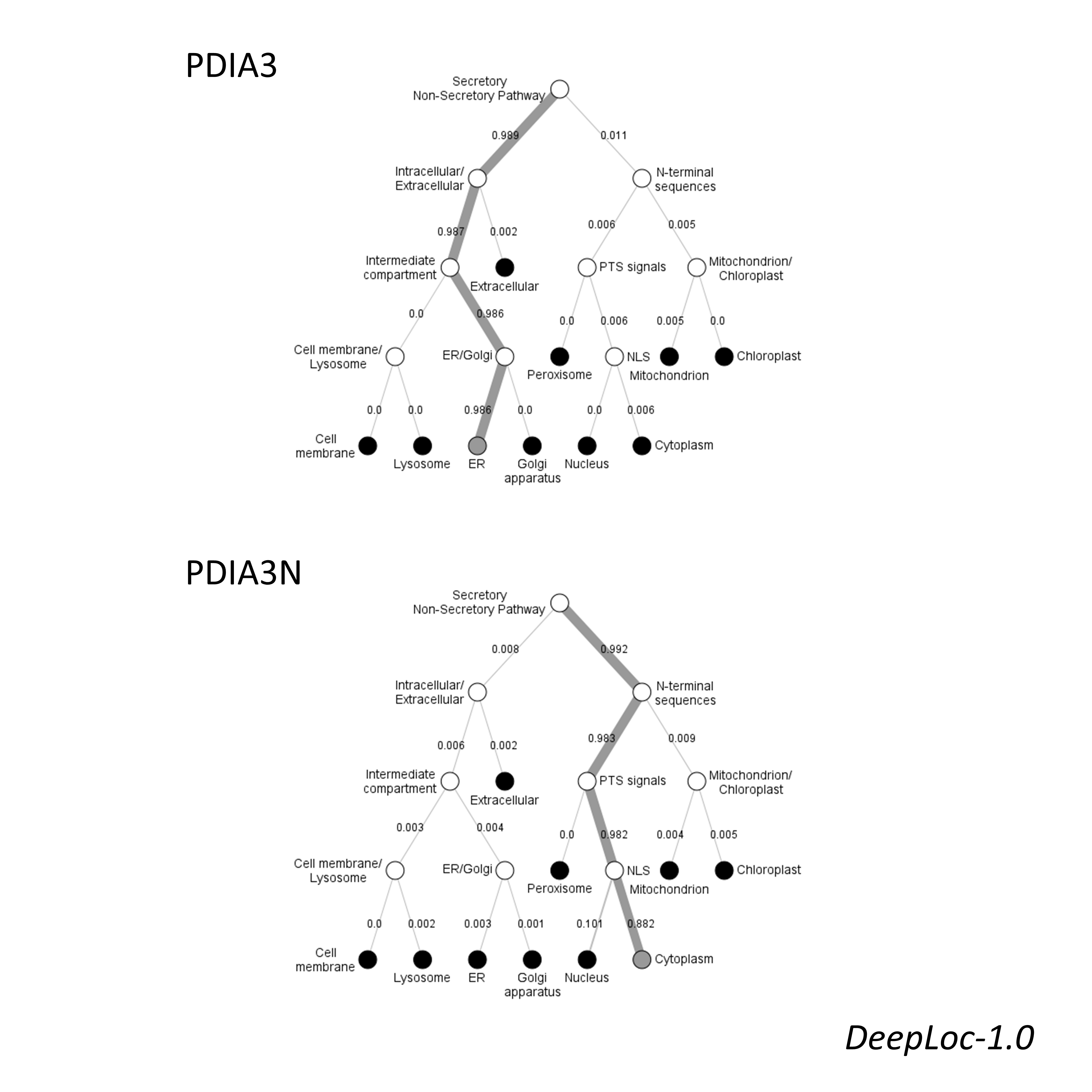

Supplement: Supplementary file 4 — Supplementary file4 (TIF 816 kb) [file 11033_2021_6277_MOESM4_ESM.tif]
