# Supplementary material for: Differential expression of protein disulfide-isomerase A3 isoforms, PDIA3 and PDIA3N, in human prostate cancer cell lines representing different stages of prostate cancer
Source: Mol Biol Rep. 2021 Mar 24;48(3):2429–36. doi: 10.1007/s11033-021-06277-1 (PMC8060222; doi:10.1007/s11033-021-06277-1)
Supplement: Supplementary file 5 — Supplementary file5 (DOCX 19 kb) [file 11033_2021_6277_MOESM5_ESM.docx]

Table 4: Results for binding analysis of 1,25(OH)2D3 with PDIA3 and PDIA3N with Autodock 4.0 software.

|  | PDIA3 | PDIA3N | VDR |
| --- | --- | --- | --- |
| *Estimated free energy of binding*  *(kcal/mol)* | -9.29^*^ | -8.21^*^ | -11.84 |
| *Estimated inhibition constant, ki*  *[temperature = 298.15 k]*  *(nanomolar; nm)* | 155.40 | 958.21 | 2.09 |
| *Final intermolecular energy (kcal/mol)* | -10.81 | -10.90 | -13.93 |
| *Vdw + hbond + desolv energy (kcal/mol)* | -10.83 | -10.84 | -13.90 |

^*^ The energy is withing the range of stating a putative binding site from the native bind in VDR. The slightly lower energy depends on that the docking simulation was performed without using flexible residues, i.e. allowing conformational changes of the receptor site. The lower energy here corresponds to a hydrogen bond between the receptor and the ligand.
